# Supplementary material for: Bovine-derived H5N1 influenza virus efficiently infects lactating swine via the mammary gland
Source: bioRxiv. 2026 Jul 18:2026.07.18.739312. Preprint. [Version 1] doi: 10.64898/2026.07.18.739312 (PMC13404955; doi:10.64898/2026.07.18.739312)
Supplement: Supplement 2 [file media-2.pdf]

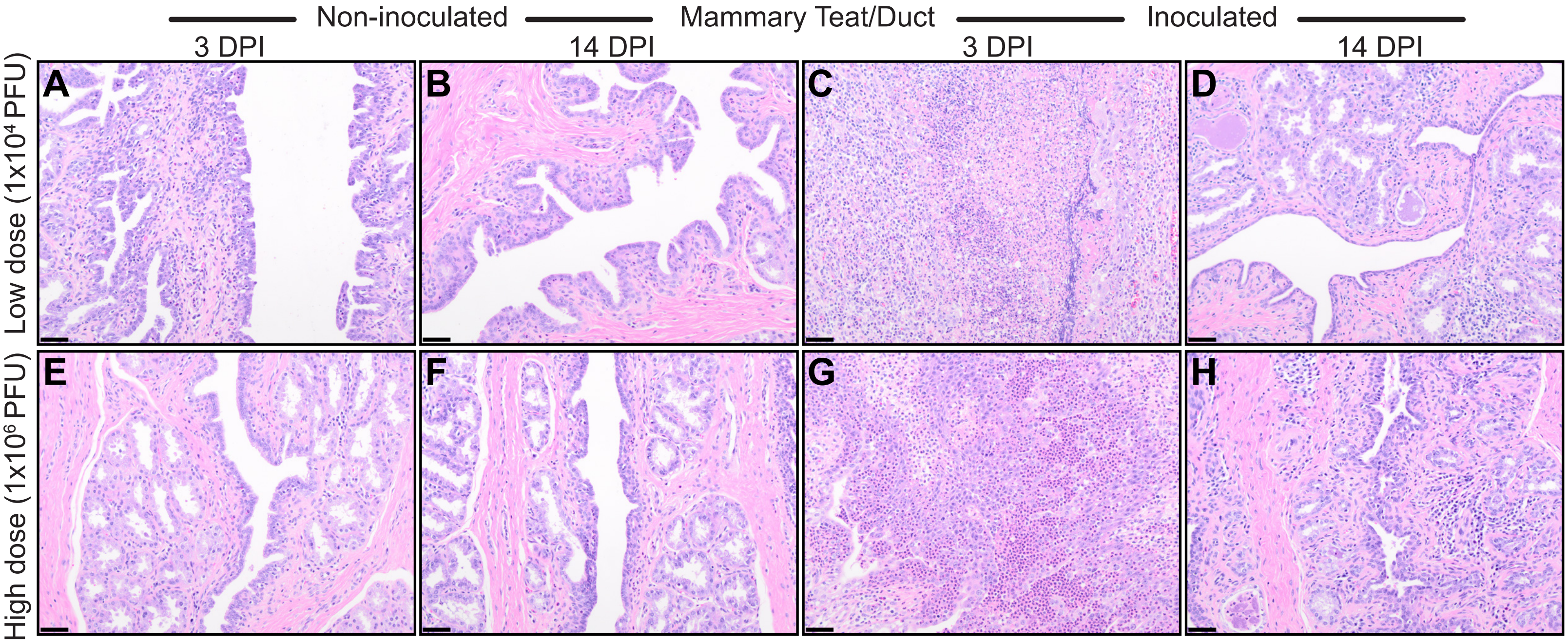

Supplementary Figure 2. Histological examination of teat and mammary duct tissue. Teat histopathology followed similar patterns as the associated uninoculated or inoculated glands with greater lesion severity. Regardless of dpi, teats from uninoculated glands (low-dose, **A-B**; high-dose, **E-F**) had minimal epithelial changes and variable interstitial infiltration by mixed mononuclear immune cells with variable numbers of eosinophils. Teats associated with inoculated glands had severe lesions at 3 dpi. Marked necrosis, loss of tissue architecture, and infiltration by myriad neutrophils were present in the low-dose teat (**C**), while the inoculated teat from the high-dose sow (**G**) had necrosis, degeneration of remaining epithelial cells, sheets of neutrophils similar to those seen in the gland, and infiltration by other inflammatory cells. At 14 dpi, the inoculated low-dose teat had epithelial attenuation/flattening and clusters of residual mononuclear inflammatory cells, particularly plasma cells (**D**). At the same timepoint, the teat from the high-dose sow (**H**) had epithelial hypertrophy and hyperplasia and larger numbers of residual lymphocytes and plasma cells with numerous eosinophils. 200x total magnification, 50 micrometer scale bars.
