# Supplementary material for: Bovine-derived H5N1 influenza virus efficiently infects lactating swine via the mammary gland
Source: bioRxiv. 2026 Jul 18:2026.07.18.739312. Preprint. [Version 1] doi: 10.64898/2026.07.18.739312 (PMC13404955; doi:10.64898/2026.07.18.739312)
Supplement: Supplement 3 [file media-3.pdf]

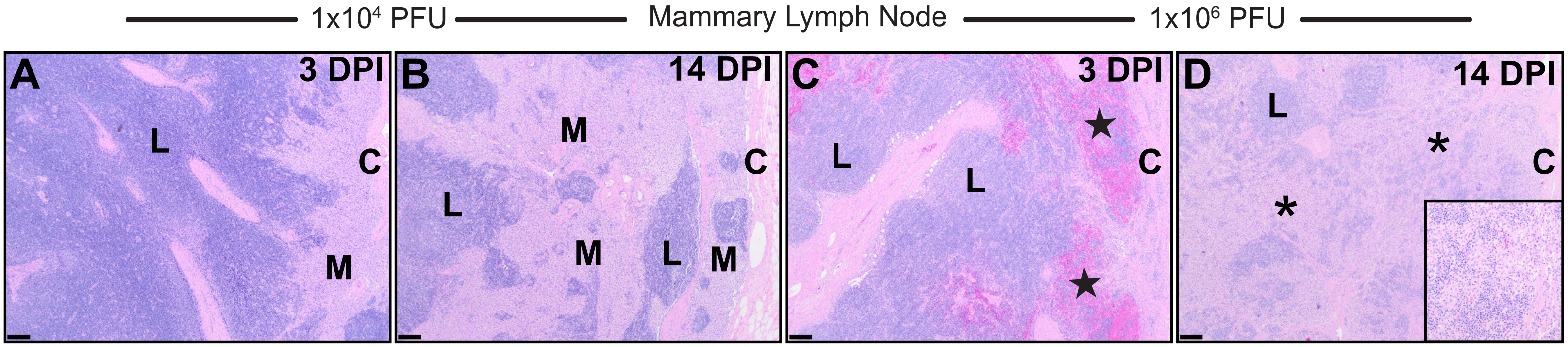

Supplementary Figure 3. Histological examination of mammary lymph nodes following intramammary inoculation. Mammary lymph nodes were relatively unaffected from low-dose sows at 3 and 14 dpi (**A** and **B**, respectively), with only an increase in cortical and medullary macrophages present at 14 dpi. Mammary lymph nodes from high-dose sows had more significant lesions including hemorrhage with hemosiderin-laden macrophages (stars) at 3 dpi (**C**) and decreased lymphocyte populations and infiltration by neutrophils, eosinophils, and increased macrophages (asterisks) at 14 dpi (**D**). L = lymphocytes; M = macrophages; C = capsule. 40x total magnification, 200 micrometer scale bars.
