# Supplementary material for: Bovine-derived H5N1 influenza virus efficiently infects lactating swine via the mammary gland
Source: bioRxiv. 2026 Jul 18:2026.07.18.739312. Preprint. [Version 1] doi: 10.64898/2026.07.18.739312 (PMC13404955; doi:10.64898/2026.07.18.739312)
Supplement: Supplement 4 [file media-4.pdf]

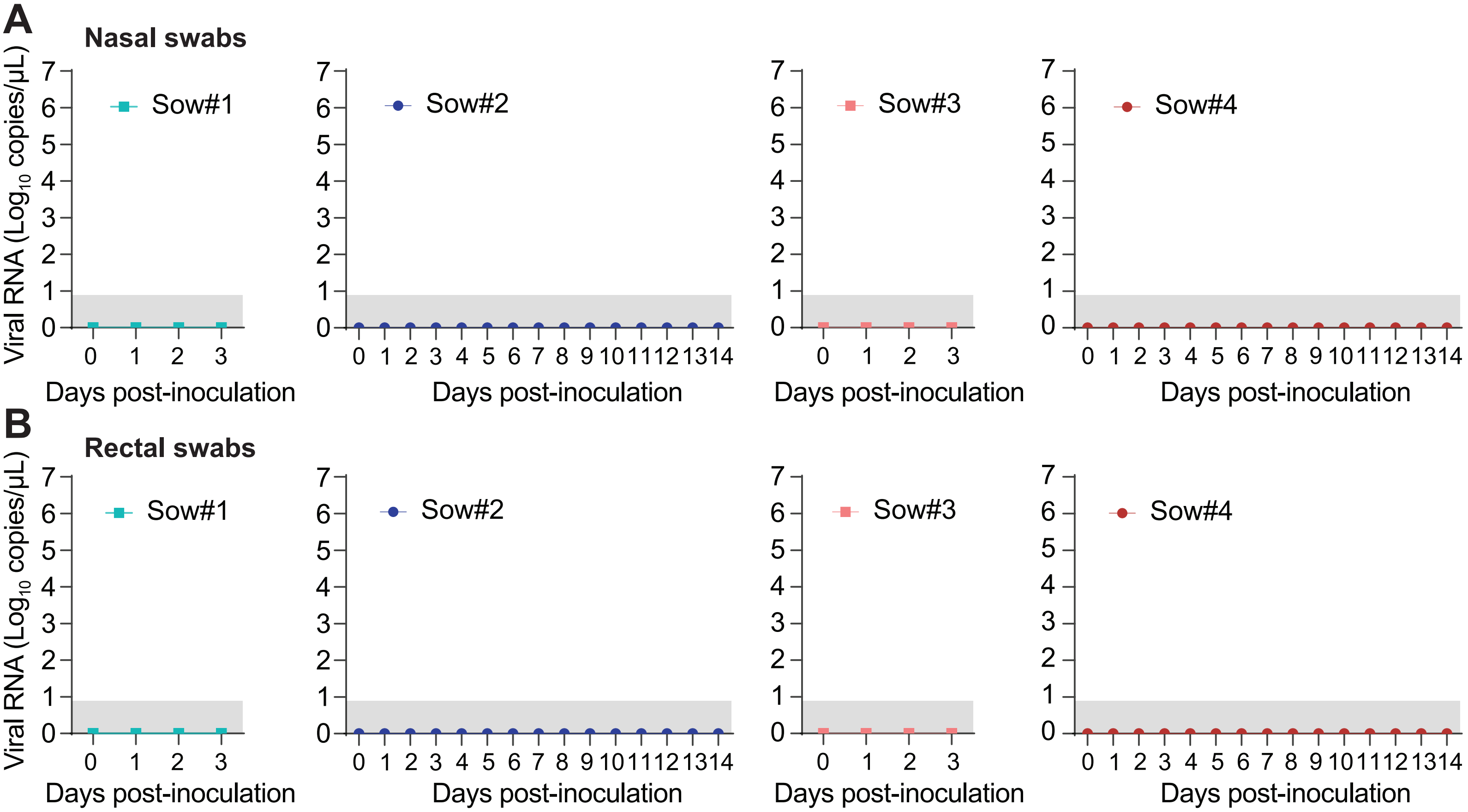

Supplementary Figure 4. No evidence of viral RNA shedding in sow nasal and rectal swabs. (**A**) Nasal and (**B**) rectal swabs were taken every day from sows, and viral RNA abundance was measured using RT-qPCR. Each data point represents Log_{10} copies of viral RNA from the indicated sow. Values plotted at 0 indicate samples with no detectable amplification signal by RT-qPCR. Shaded regions represent the limit of detection for the assay.
