# Supplementary material for: Bovine-derived H5N1 influenza virus efficiently infects lactating swine via the mammary gland
Source: bioRxiv. 2026 Jul 18:2026.07.18.739312. Preprint. [Version 1] doi: 10.64898/2026.07.18.739312 (PMC13404955; doi:10.64898/2026.07.18.739312)
Supplement: Supplement 6 [file media-6.pdf]

Supplemental Table 2. Observational findings of milk in lactating sows following intramammary inoculation with influenza A(H5N1) virus D1.1 genotype

Sow ID	[a]dpi	[b]Milk color and consistency change													
		Inoculated mammary glands							Non-inoculated mammary glands						
		L1	L3	L5	L7	R2	R4	R6	L2	L4	L6	R1	R3	R5	R7
Sow#4	0	-	-	-	-	-	-	-	-	NA	-	-	-	-	-
	1	NA	-	-	-	-	-	-	-	NA	-	-	-	NA	-
	2	-	-	-	-	-	-	-	-	-	-	-	-	-	-
	3	-	-	-	-	+	-	+	-	-	-	-	-	-	-
	4	+	+	+	-	+	-	+	-	-	-	-	-	-	-
	5	+	-	+	+	+	-	-	-	-	-	-	-	-	-
	6	+	-	+	+	+	-	+	-	-	-	-	-	-	-
	7	+	+	+	+	+	+	+	-	-	-	-	-	-	-
	8	+	-	+	+	+	+	+	-	-	-	-	-	-	-
	9	-	-	-	+	+	-	-	-	-	-	-	-	-	-
	10	-	-	-	-	+	-	-	-	-	-	-	-	-	-
	11	-	-	-	-	+	-	-	-	-	-	-	-	-	-
	12	-	-	-	-	+	-	-	-	-	-	-	-	-	-
	13	-	-	-	-	+	-	-	-	-	-	-	-	-	-
	14	-	-	-	-	+	-	-	-	-	-	-	-	-	-

[a]dpi: days post-inoculation.
[b]Daily milk samples from each mammary gland were examined for changes in color and consistency. (+): Observed changes in milk appearance, including yellowish discoloration, thickened consistency, or the presence of trace blood. (-): Milk exhibiting typical, unchanged color and texture. NA (Not Applicable): Not applicable as milk yield was too low or absent to permit a visual assessment.
